# Functional role of GATA3 and CDX2 in lineage specification during bovine early embryonic development

**DOI:** 10.1530/REP-22-0269

**Published:** 2023-02-08

**Authors:** Yan Shi, Bingjie Hu, Zizengchen Wang, Xiaotong Wu, Lei Luo, Shuang Li, Shaohua Wang, Kun Zhang, Huanan Wang

**Affiliations:** 1 Laboratory of Mammalian Molecular Embryology, College of Animal Sciences, Zhejiang University, Hangzhou, China

## Abstract

**In brief:**

The lineage specification during early embryonic development in cattle remains largely elusive. The present study determines the effects of trophectoderm-associated factors GATA3 and CDX2 on lineage specification during bovine early embryonic development.

**Abstract:**

Current understandings of the initiation of the trophectoderm (TE) program during mammalian embryonic development lack evidence of how TE-associated factors such as GATA3 and CDX2 participate in bovine lineage specification. In this study, we describe the effects of TE-associated factors on the expression of lineage specification marker genes such as *SOX2, OCT4, NANOG, GATA6,* and *SOX17*, by using cytosine base editor system. We successfully knockout *GATA3* or *CDX2* in bovine embryos with a robust efficiency. However, GATA3 or CDX2 deletion does not affect the developmental potential of embryos to reach the blastocyst stage. Interestingly, GATA3 deletion downregulates the NANOG expression in bovine blastocysts. Further analysis of the mosaic embryos shows that GATA3 is required for NANOG in the TE of bovine blastocysts. Single blastocyst RNA-seq analysis reveals that GATA3 deletion disrupts the transcriptome in bovine blastocysts. Altogether, we propose that GATA3 plays an important role in maintaining TE lineage program in bovine embryos and the functional role of GATA3 is species-specific.

## Introduction

After fertilization, embryos can develop through continuous cleavage into fluid-filled blastocysts consisting of the trophectoderm (TE) and inner cell mass (ICM). The formation of a blastocyst commonly involves two rounds of cell lineage segregation (Karasek *et al.* 2020). During the polarization in mouse morula, atypical protein kinase C complex can sequester, angiomotin (AMOT) at the apical domain thereby keeping the inactivation state of Hippo signaling pathway to reinforce the expression of TE-associated genes such as *Cdx2* and *Gata3* (Plusa *et al.* 2005, Nishioka *et al.* 2009).

In mice, CDX2 and OCT4 mutually repress each other in TE and ICM, respectively, which signals the completion of the first lineage specification (Niwa *et al.* 2005). Subsequently, ICM can give rise to primitive endoderm (PE) and epiblast (EPI) during the second cell lineage decision. PE marked by GATA6 and SOX17 would develop into extra-embryonic tissues and EPI marked by NANOG would contribute to the embryo proper (Zhu & Zernicka-Goetz 2020, Yildirim *et al.* 2021). Understanding the molecular machinery of lineage specification is important for proper embryonic development and stem cell derivation.

TE-associated genes such as *Tead4*, *Gata3,* and *Cdx2* play an important role in the regulation of early lineage development. TEAD4 acts as a coactivator of YAP1 to regulate GATA3 and CDX2 via the Hippo signaling pathway in mouse embryos (Yagi *et al.* 2007, Nishioka *et al.* 2009). CDX2 is a downstream transcription factor of TEAD4 and is essential for TE lineage commitment. Indeed, CDX2 deletion impairs the TE epithelial integrity and results in the collapse of the blastocyst and the failure to implant in mouse embryos (Strumpf *et al.* 2005). Moreover, the absence of *Cdx2* results in the expression of *Oct4* and *Nanog* in the outer cells of mouse blastocysts. ChIP-seq results in mouse TE stem cells reveal that *Oct4* and *Nanog* are direct targets of *Cdx2* (Huang *et al.* 2017). Forced repression of OCT4 can induce mouse embryonic stem cells to differentiate into TE lineage (Niwa *et al.* 2005). Thus, there is reciprocal inhibition between TE and ICM lineage-specific transcription factors in mice.

As another downstream regulator of TEAD4, GATA3 is present exclusively in the TE but not the ICM in mouse embryos. *Gata3* knockout (KO) embryos are arrested at day 11 post coitum (Home *et al.* 2017). Meanwhile, GATA3 directly regulates *Cdx2* transcription and disrupts the morula to blastocyst transition in mice (Home *et al.* 2009). However, if GATA3 regulated ICM-specific transcriptional factors has yet to be determined.

Although the molecular machinery of lineage specification has been extensively studied in mouse embryos, the lineage segregation of bovine embryos remains unclear largely due to technical limitation. Recently, we have successfully implemented cytosine base editors (CBE) system in bovine embryos to achieve genome editing (Luo *et al.* 2022), which is a robust tool to interrogate gene functions.

Here, we first validated the effectiveness of the base editing system to knockout *GATA3* or *CDX2* in bovine embryos. We noticed that the developmental capability of embryos to form blastocyst is not changed after GATA3 or CDX2 deletion. Surprisingly, the NANOG level is reduced in GATA3 KO blastocysts. RNA-seq results show that the transcriptome is also disrupted in bovine blastocysts. In summary, these data suggest that GATA3 is an important player involved in maintaining the TE lineage program in bovine embryos.

## Materials and methods

### Materials

All chemicals and reagents were commercially obtained from Sigma unless stated elsewhere.

### *In vitro* production of bovine embryos

*In vitro* production of bovine embryos, including *in vitro* maturation (IVM), *in vitro* fertilization (IVF), and *in vitro* culture (IVC), was performed as published before (Cui *et al.* 2016, Li *et al.* 2021, Shi *et al.* 2021). Briefly, cumulus–oocyte complexes (COCs) containing intact cumulus cells were collected from bovine ovaries obtained from a local abattoir and these COCs were matured in the IVM medium at 38.5°C under 5% CO_2_ in humidified air for 22–24 h. The IVM medium consists of 10% fetal bovine serum (FBS; Gibco-BRL), 1 IU/mL follicle-stimulating hormone (Sansheng Biological Technology, Ningbo, China), 0.1 IU/mL luteinizing hormone (Solarbio, Beijing, China), 1 mM sodium pyruvate (Thermo Fisher Scientific), 2.5 mM GlutaMAX™ (Thermo Fisher Scientific), and 10 μg/mL gentamicin in Medium-199 (M4530). After maturation, COCs (60–100 COCs per well in 4-well plates) were then incubated with spermatozoa (1 × 10^6^–5 × 10^6^) purified from frozen-thawed semen using a percoll gradient in BO-IVF medium (IVF Bioscience, Falmouth, Cornwall, UK). The IVF condition was 38.5°C under 5% CO_2_ for 9–12 h. Putative zygotes were then removed from cumulus cells by pipetting up and down in Medium-199 (M7528) supplemented with 2% FBS (Gibco-BRL). Embryos were incubated in BO-IVC medium (IVF Bioscience) at 38.5°C under 5% CO_2_ in humidified air until use.

### sgRNA design, synthesis, and plasmid construction

BE-Designer online software (http://www.rgenome.net) was used to design sgRNAs. sgRNA sequences with appropriate GC content and low probability for off-targeting were selected to target the coding region of the gene of interest. The sticky end of BpiI: 5’-3’ CACC and 5’-3’ AAAC were added to the 5’ ends of the sense and antisense strand, respectively (Supplementary Table 1, see section on supplementary materials given at the end of this article). The DNA sequences were synthesized by Sangon Co., Ltd (Shanghai, China). Then, sgRNA DNA oligos were annealed and cloned into a PX458 vector containing BpiI restriction sites with a T7 promoter.

### *In vitro* transcription

*In vitro* transcription was performed as published previously (Luo *et al.* 2022). BE3 plasmids were linearized with NotI and underwent *in vitro* transcription using mMESSEAGE mMACHINE T7 kit (Invitrogen, Thermo Fisher Scientific) and finally purified by LiCl precipitation. sgRNAs were amplified and transcribed *in vitro* using MEGAshortscript T7 High Yield Transcription Kit (Invitrogen, Thermo Fisher Scientific) according to the manufacturer’s instructions. Primers are listed in Supplementary Table 2. After transcription, sgRNAs were purified by ethanol precipitation.

### Microinjection of base editor mRNA

To knockout the gene of interest, a 10–20 pL mixture of 100 ng/µL sgRNA and 200 ng/µL BE3 mRNA were microinjected into putative zygotes 10 h after IVF by using a micromanipulator (TransferMan, Eppendorf, Germany). In the control group, embryos were injected with the same amount of BE3 mRNA without sgRNA. To maximize the editing efficiency of the gene of interest, a cocktail of two or three sgRNAs was microinjected together with BE3 mRNA. Each sgRNA was kept at the same concentration (100 ng/µL).

### Single embryo genotyping

After immunostaining, each embryo was collected individually to perform genotyping. Genomic DNA was isolated using an embryo lysis buffer (40 nM Tris-HCl, 1% Triton X-100, 1% NP-40, and 0.4 ng/mL proteinase K) at 55°C for 1 h and 95°C for 10 min. Nested PCR was performed and amplicons were then subject to Sanger sequencing. For nested PCR, two rounds of PCR were performed by using primeSTAR HS DNA Polymerase (Takara, Cat. #R040A). The PCR condition was: 98°C for 2 min followed by 35 cycles of 98°C for 10 s, 60°C for 5 s, 72°C for 1.5 min, and a final 5 min step at 72°C. All primers used are listed in Supplementary Table 3.

### Immunofluorescence

Early embryos were rinsed three times with 0.1% PBS/polyvinylpyrrolidone and fixed with 4% paraformaldehyde in PBS for 30 min, permeabilized with 0.5% Triton X-100/PBS for 40 min. Fixed samples were then blocked for 2 h with a buffer containing 10% FBS and 0.1% Triton X-100/PBS. Samples were incubated with primary antibodies overnight at 4°C, and embryos were then treated with secondary antibodies for 2 h. All antibodies were both confirmed in bovine embryos by our previous work and other labs, including CDX2, GATA6, and NANOG (Wooldridge & Ealy 2021); GATA3 (Gerri *et al.* 2020); and SOX2 and SOX17 (Luo *et al.* 2022). We have also validated the specificity of OCT4 and SOX2 using KO strategy in bovine embryos (Luo *et al.* 2022). Nuclear DNA was counterstained by DAPI for 20 min. Samples were mounted and observed with either an inverted epifluorescence microscope (Nikon) or a Zeiss LSM880 confocal microscope system (Zeiss). For confocal microscopy, Z-stacks were imaged at 5 μm intervals between optical sections. Stacks were projected by maximum intensity to display signals of all blastomeres in one image.

The fluorescent intensity was analyzed using Image J as described previously (Shi *et al.* 2021). Briefly, the nuclear region was encircled based on the DAPI signal and the intensity measured. The same region was moved to the cytoplasm area and background intensity was obtained. The specific signal was calculated by subtracting the cytoplasmic intensity from the nuclear intensity. Finally, the data were normalized to the relative channels in the control groups. All antibody information is shown in Supplementary Table 4.

### Single blastocyst RNA-seq

Single blastocysts from WT and KO group were collected on day 7. The zona pellucida of blastocysts was discarded with 0.5% pronase E. The RNA-seq libraries were constructed according to Smart-seq2 procedure as previously described (Picelli *et al.* 2014). In brief, polyadenylated RNAs were captured and reverse transcribed with an Oligo(dT) primer, then the cDNA was pre-amplified using KAPA HiFi HotStart ReadyMix (kk2601). Pre-amplified cDNA was purified with Ampure XP beads (1:1 ratio) and fragmented by Tn5 enzyme (Vazyme, TD502). PCR amplification for 15–18 cycles was performed to prepare sequencing libraries, which were subject to paired-end 150 bp sequencing on a NovaSeq (Illumina) platform by Novogene. The raw sequencing reads were trimmed with Trimmomatic (version 0.39) (Bolger *et al.* 2014) to generate clean data and mapped to ARS-UCD1.2 with Hisat2 (version 2.1.0) (Kim *et al.* 2015). The raw counts were calculated with featureCounts (version 1.6.3) (Liao *et al.* 2014) and underwent differential expression analysis using DESeq2 (Love *et al.* 2014). The differentially expressed WT and KO gene groups were identified using an false discovery rate (FDR)-adjusted *P*-value (*P*_adj_) less than 0.05. Foldchange ≥1.5 or ≤0.6. Fragments per kilobase million (FPKM) for each sample was calculated with Cufflinks (Trapnell *et al.* 2012) for heatmap visualization, and heatmaps were generated using a pheatmap package in R. Gene ontology analysis was performed with the Database for Annotation, Visualization and Integrated Discovery (Huang da *et al.* 2009*a*,*b*). All RNA-seq files are available from the Gene Expression Omnibus database (accession number GSE216123).

### Statistical analysis

All experiments were replicated at least three times unless stated. Two-tailed unpaired Student *t*-tests were used to compare differences between the two groups. The graphs were constructed by GraphPad Prism 8.0 (GraphPad Software). Asterisks refer to significant differences (*P < 0.05; **P < 0.05; ***P < 0.001).

## Results

### Disrupting *GATA3* and *CDX2* with cytosine base editor in bovine embryos

**Figure 1:**
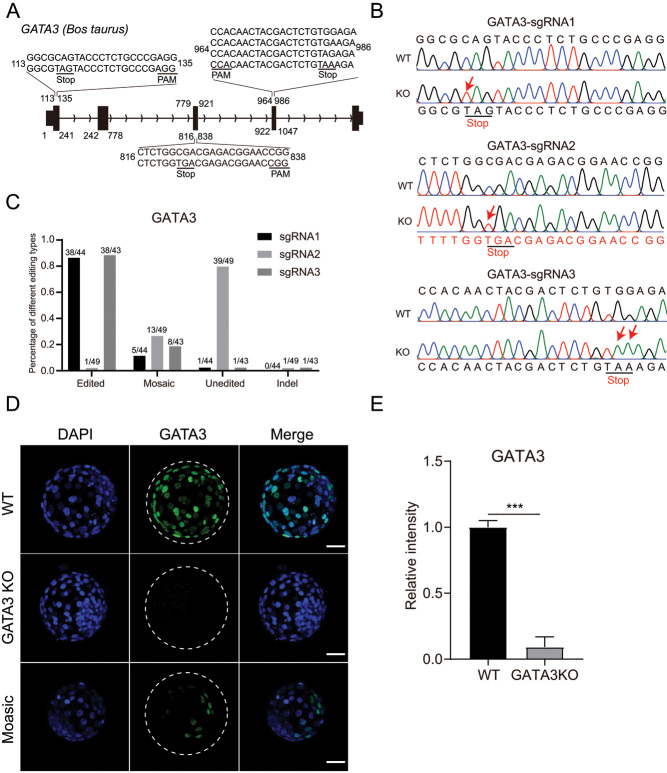
The construction of GATA3 KO model in bovine embryos. (A) Three sgRNAs were designed to target exons 1, 3, and 4 of *GATA3*. The red letters represent potential sites for introducing premature stop codons. (B) Representative Sanger sequencing results. The red arrows represent edited sites. (C) Statistical analysis of the editing efficiency in different editing types for GATA3 sgRNA1 (44 embryos), sgRNA2 (49 embryos), and sgRNA3 (43 embryos). (D) Immunostaining validation for GATA3 KO at blastocysts stage (three replicates of 7–9 blastocysts per group). Green: GATA3; Scale bar = 50 μm. (E) Statistical analysis of GATA3 relative intensity in bovine embryos (three replicates of 7–9 blastocysts per group). Asterisks refer to significant differences (*P < 0.05; **P <0.05; *** *P* < 0.001).

To explore the effects of TE-associated factors (GATA3 and CDX2) on lineage specification in bovine early embryos, we designed sgRNAs to disrupt *CDX2* and *GATA3*, respectively. *CDX2* sgRNAs have been validated recently (Luo *et al.* 2022). For *GATA3,* three sgRNAs were designed to target exons 1, 3, and 4, respectively (Fig. 1A). To maximize editing efficiency, we microinjected sgRNAs cocktails and CBE mRNA into putative zygotes. At day 7 after fertilization, bovine embryos were collected for performing immunostaining and genotyping. Sanger sequencing results show that premature stop codons could be successfully introduced by injection of CBE components (Fig. 1B).

Furthermore, results show that the edited efficiency of sgRNA1, sgRNA2, and sgRNA3 for *GATA3* is 86.36%, 2.04%, and 88.37%, respectively (Fig. 1C). Overall, premature stop codons were successfully introduced into 94.12% (48/51) of bovine embryos. Meanwhile, it was confirmed that GATA3 can be efficiently depleted by immunostaining analysis of the corresponding embryo (Fig. 1D and E). Thus, we establish a robust model to interrogate the functional role of GATA3 in bovine embryos.

### CDX2 is dispensable for bovine lineage specification

CDX2 plays a critical role in TE lineage development in mice. It is established that CDX2 and OCT4 reciprocally inhibit each other in TE and ICM, respectively, in mouse late blastocysts (Niwa *et al.* 2005). The depletion of CDX2 also results in the failure to downregulate NANOG in the outer cells of the blastocyst and increases the incidence of cell death (Strumpf *et al.* 2005). However, the functional role of CDX2 in bovine early embryonic development remains poorly known.

**Figure 2:**
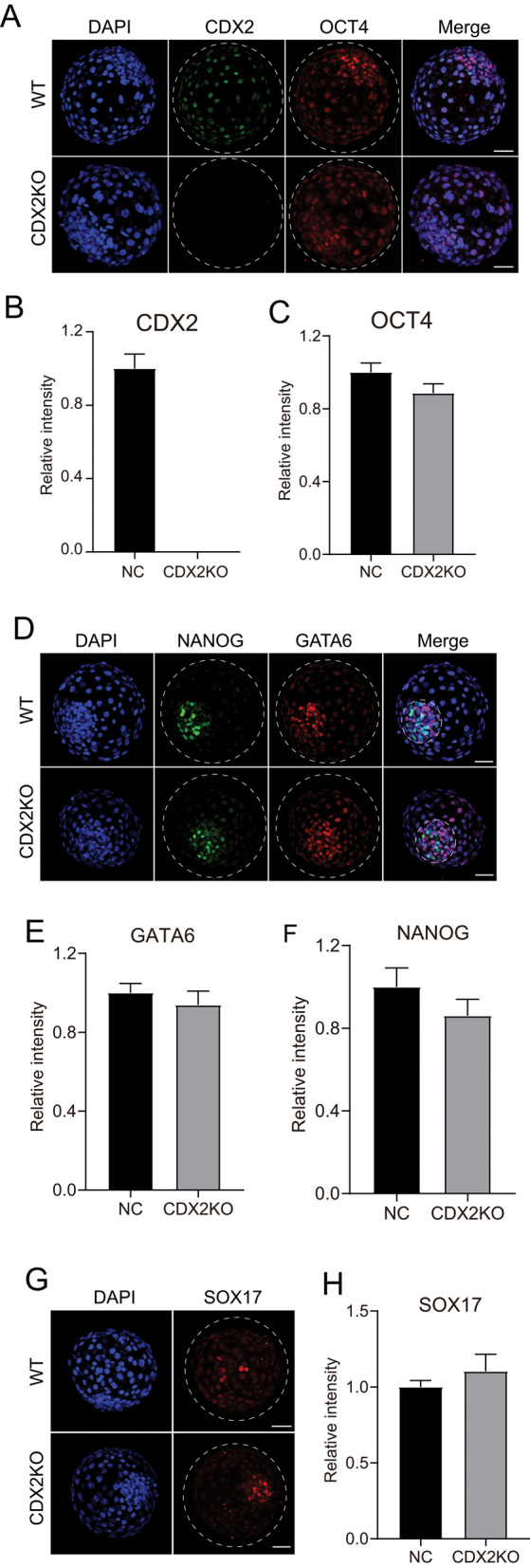
CDX2 is dispensable for bovine lineage specification. (A) Immunofluorescence detection of CDX2 and OCT4 in WT and CDX2 KO blastocysts at day 8. Green: CDX2 protein; Red: OCT4 protein; Blue: DAPI (nuclei). The experiment was independently replicated four times with 4–8 blastocysts per group analyzed. Scale bar = 50 μm. (B and C) Statistical analysis of CDX2 and OCT4 relative intensity. (D) Immunofluorescence analysis of NANOG and GATA6 expression and allocation between WT and CDX2 KO groups (three replicates of 3–8 blastocysts per group were analyzed). Green: NANOG protein; Red: GATA6 protein; Blue: DAPI (nuclei). Scale bar = 50 μm. (E and F) Statistical analysis of GATA6 and NANOG levels. (G) Immunostaining analysis of SOX17 in WT and CDX2 KO blastocysts at day 8 (three replicates of 6–10 blastocysts per group were analyzed). Red: SOX17 protein; Blue: DAPI (nuclei). Scale bar = 50 μm. (H) Statistical analysis of SOX17 signal intensity. Asterisks refer to significant differences (*P < 0.05; **P <0.05; ***:*P* < 0.001).

We then adopted the CBE system to evaluate the developmental and molecular consequence of CDX2 KO in bovine embryos. Results show no obvious difference in OCT4 expression in CDX2 null embryos compared to WT embryos (Fig. 2A). Unlike mouse embryos, the localization of NANOG, GATA6, and SOX17 was not completely restricted into the ICM region at late blastocyst stage in cattle (Bessonnard *et al.* 2014). In addition, we found no significant change in the localization and signal intensity of NANOG, GATA6, and SOX17 in CDX2-depleted blastocysts (Fig. 2D, E, F, G and H). Altogether, these results reveal that CDX2 is dispensable for bovine lineage specification.

### GATA3 is required for NANOG expression in TE

**Figure 3:**
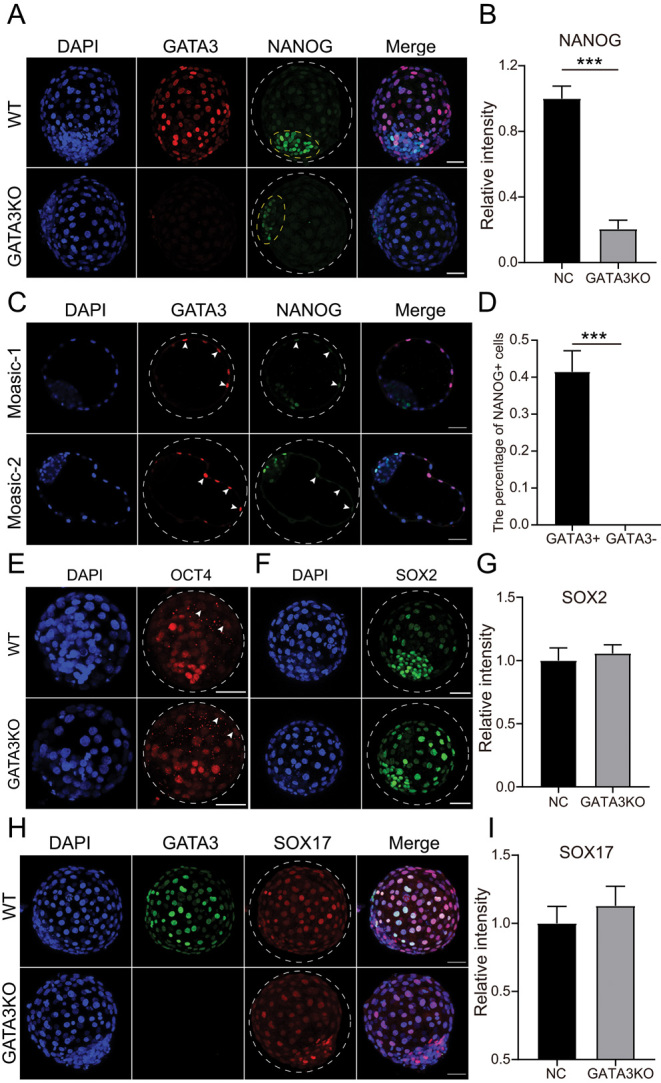
GATA3 is required for NANOG expression in the TE. (A) Immunostaining analysis of NANOG and GATA3 in WT and GATA3 KO blastocysts at day 8. Green: NANOG protein; Red: GATA3 protein; Blue: DAPI (nuclei). The experiment was independently replicated three times with 6–9 blastocysts per group analyzed. Scale bar = 50 μm. The yellow circle represents the ICM region. (B) Statistical analysis shows that NANOG is decreased in GATA3 KO group. (C) Immunostaining analysis of NANOG levels in GATA3− TE cells relative to GATA3+ TE cells. Green: NANOG protein; Red: GATA3 protein; Scale bar = 50 μm. White arrows indicate TE cells expressed both GATA3 and NANOG. (D) Statistical analysis of the percentage of NANOG+ cells in GATA3− TE cells relative to GATA3+ TE cells. *n* = 8. (E) Immunostaining detection of OCT4 levels. *n* = 7. White arrows point to TE cells expressed OCT4. (F) Immunostaining analysis of SOX2 in WT and GATA3 KO blastocysts (three replicates of 4–5 blastocysts per group were analyzed). Green: SOX2 protein; Blue: DAPI (nuclei). Scale bar = 50 μm. (G) Statistical analysis of SOX2 signal intensity between WT and GATA3 KO groups. (H) Immunostaining analysis of SOX17 and GATA3 in WT and GATA3 KO blastocysts (two replicates of 6–7 blastocysts per group were analyzed). Green: GATA3 protein; Red: SOX17 protein; Blue: DAPI (nuclei). Scale bar = 50 μm. (I) Statistical analysis of SOX17 signal intensity between WT and GATA3 KO groups. Asterisks refer to significant differences (*P < 0.05; **P < 0.05; *** *P* < 0.001).

GATA3 and YAP1 co-localized in outer cells at the morula stage in bovine embryos (Gerri *et al.* 2020), suggesting that a close association between GATA3 and TE lineage fate. However, it remains unclear if GATA3 inhibits the expression of the pluripotency gene. Therefore, we looked into the expression levels of SOX2, NANOG, and SOX17 in GATA3 KO blastocysts. Immunostaining results show that GATA3 KO did not affect the expression of SOX2 and SOX17 (Fig. 3G and I). In WT bovine blastocyst, NANOG is predominantly localized in the ICM. However, NANOG could also be detected in the TE. Surprisingly, the signal intensity of NANOG is greatly reduced in the ICM. Moreover, NANOG is barely seen in the TE of GATA3 KO blastocysts (Fig. 3A).

To investigate if GATA3 directly affected the NANOG expression in TE, we constructed GATA3 mosaic embryos and counted the number of NANOG+ cells out of the number of GATA3+ or GATA3− cells in the TE. Results clearly show there is 40% of GATA3+ cells are also NANOG+, whereas no NANOG+ cells were observed in GATA3− TE cells (Fig. 3D). Thus, GATA3 is required for correct NANOG expression pattern in the TE of bovine blastocysts.

Recent studies reveal that OCT4 is required for NANOG expression in bovine embryos (Simmet *et al.* 2018). Thus, we sought to determine whether GATA3 regulates NANOG expression through OCT4. Immunostaining results show no obvious change in OCT4 level in the TE of GATA3 KO blastocysts. In conclusion, GATA3 plays an important role in the regulation of NANOG expression in the TE of bovine blastocysts.

### GATA3 and CDX2 function independently on bovine embryos

**Figure 4:**
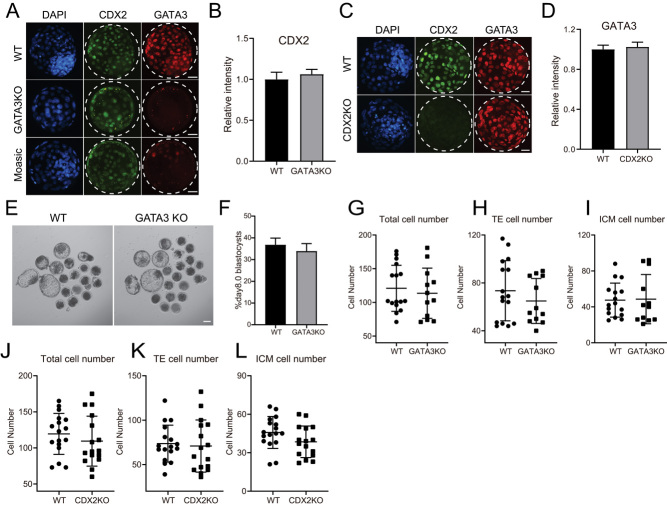
GATA3 and CDX2 function independently in bovine embryos. (A) Immunostaining analysis of CDX2 and GATA3 in WT and GATA3 KO blastocysts (three replicates of 4–7 blastocysts per group were analyzed). Green: CDX2 protein; Red: GATA3 protein; Blue: DAPI (nuclei). Scale bar = 50 μm. (B) Statistical analysis of CDX2 signal intensity in the GATA3 KO group relative to the WT group. (C) Immunostaining analysis of CDX2 and GATA3 in WT and CDX2 KO blastocysts (three replicates of 6–10 blastocysts per group were analyzed). Green: CDX2 protein; Red: GATA3 protein; Blue: DAPI (nuclei). Scale bar = 50 μm. (D) Statistical analysis of GATA3 relative signal intensity. (E and F) GATA3 KO do not affect the blastocyst rate (ten replicates of 20–25 embryos per group). Scale bar = 100 μm. (G, H, and I) Cell counting analysis of total cells (DAPI), TE cells (CDX2+ cells) and ICM cells (CDX2− cells) in WT and GATA3 KO blastocysts at day 8. (J, K, and L) Statistical analysis of the number of total cells (DAPI), TE cells (GATA3+ cells) and ICM cells (GATA3− cells) in WT and CDX2 KO blastocysts at day 8. Asterisks refer to significant differences (*P < 0.05; **P <0.05; ***P < 0.001).

Next, we wondered whether GATA3 interacts with CDX2 in bovine embryos. Results show that the localization and signal intensity of CDX2 are not changed in GATA3 KO embryos compared to controls (Fig. 4B and D). Furthermore, the developmental capability of GATA3 KO and CDX2 KO in bovine embryos was monitored. On day 8, we used CDX2 as a marker to distinguish the TE and ICM cells for GATA3 KO embryos and GATA3 as a marker to count the TE and ICM cells for CDX2 KO embryos. Results show the normal developmental potential of GATA3 KO bovine embryos to reach the blastocyst stage (Fig. 4E and F) and normal cell numbers of the TE and ICM in the blastocysts (Fig. 4G, H and I). Similarly, the total cell number and the number of TE and ICM numbers are normal in CDX2 KO bovine embryos (Fig. 4J, K and L). Collectively, these results suggest that CDX2 and GATA3 function independently on bovine embryos.

### Functional role of GATA3 in bovine early embryonic development

To dissect the molecular consequence of GATA3 or CDX2 KO in bovine embryos, a single blastocyst RNA-seq was performed. Base editing in *GATA3* and *CDX2* was confirmed by cDNA Sanger sequencing (Supplementary Fig. 1B and C). Interestingly, there is no obvious change in the level of GATA3 and CDX2 transcripts in GATA3 or CDX2 KO embryos, respectively. It is likely that the premature stop codon that created by CBE would disrupt the open reading frame of GATA3 and CDX2 and not affect the transcription itself.

**Figure 5:**
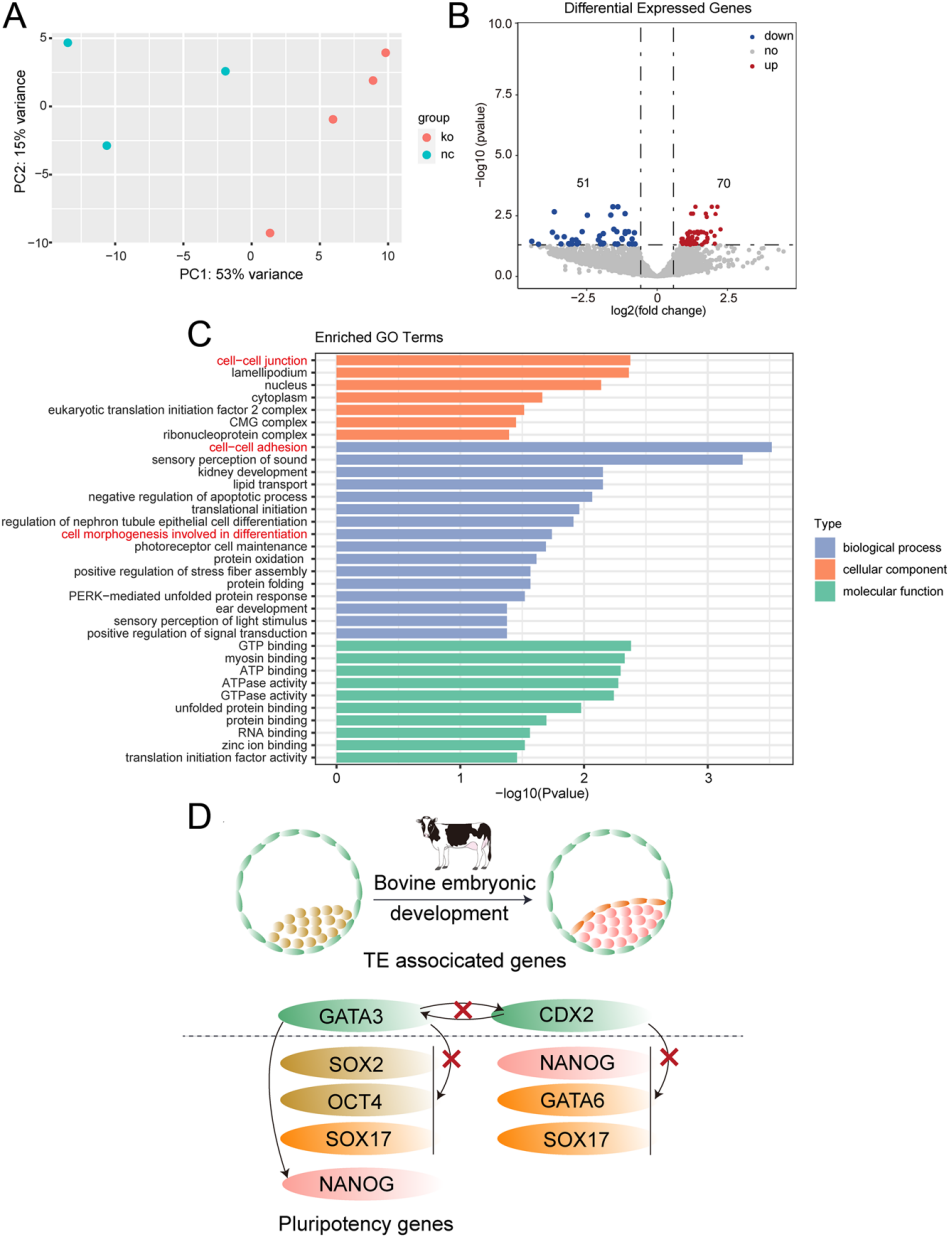
GATA3 deletion disrupts the transcriptome in bovine blastocysts. (A) Principal component analysis (PCA) shows a high correlation among samples in WT and GATA3 KO groups, respectively. (B) Volcano plot depicting differentially expressed genes, among which 70 are upregulated and 51 are downregulated (Fold change ≥1.5 or ≤0.6; *P*_adj_ ≤ 0.05). (C) GO terms analysis showing differential expression of genes enriched in the pathway of cell–cell adhesion, cell–cell junction, and cell morphogenesis involved in differentiation. (D) Summary and working model of a functional relationship between TE-associated genes (*GATA3 and CDX2*) and pluripotency genes (*SOX2, OCT4, NANOG, GATA6, and SOX17*) in bovine blastocysts. Arrows denote that ‘A’ affects ‘B’, and ‘X’ indicates that ‘A’ does not affect ‘B’.

RNA-seq analysis showed the transcriptome of CDX2 KO embryos clusters with one of the WT embryos (Supplementary Fig. 1D). In addition, there is a total of 121 differentially expressed genes (DEGs, threshold: Fold changes (FC) ≥2 or ≤0.5, FDR adjusted *P*-value ≤ 0.05), among which 70 are upregulated and 51 are downregulated in GATA3 KO embryos (Fig. 5B). GO analysis shows the top GO terms enriched in DEGs include cell–cell adhesion, cell–cell junction, and cell morphogenesis involved in differentiation (Fig. 5C).

## Discussion

The establishment of the first lineage specification is generally conserved in the mammalian embryo. However, the regulatory mechanism of transcriptional factors may vary slightly among species. In particular, the relationship between members of transcription factor networks controlling lineage development in bovine embryos remains largely unclear. One of the limiting factors is the lack of a technical approach to efficiently knockout the gene of interest. Here, we successfully delete GATA3 and CDX2 by using the CBE system with robust efficiency. GATA3 deletion does not affect the distribution and expression level of OCT4. Moreover, GATA3 and CDX2 do not affect each other in bovine embryos. However, NANOG is greatly reduced in bovine blastocysts upon GATA3 deletion. Last but not least, the transcriptome of GATA3 KO embryos is disturbed.

Previous studies have demonstrated reciprocal repression between TE and ICM lineage factors in mice. For example, TE-specific CDX2 and ICM-specific OCT4 repress each other in mouse blastocysts (Niwa *et al.* 2005). In accordance, another study shows that *Oct4* and *Nanog* are expressed in the out cells of *Cdx2* KO blastocysts (Strumpf *et al.* 2005). In the present study, we found that CDX2 null embryos show normal OCT4 expression, which is consistent with the result of CDX2 knockdown study (Berg *et al.* 2011). Thus, by comparing the data in mice (Niwa *et al.* 2005), these results suggest the relationship between CDX2 and OCT4 is species-specific.

NANOG and GATA6 are well-established markers for EPI and PE, respectively (Frankenberg *et al.* 2011). We found a typical salt and pepper pattern of NANOG and GATA6 localization in CDX2 KO bovine blastocysts. SOX17 is also not been affected. Thus, these results confirm that CDX2 is dispensable for the second lineage specification that generates EPI and PE.

In normal bovine embryos, NANOG is readily detected in the ICM while it is also found in the TE with a lower level at the late blastocyst stage. Interestingly, we noticed that GATA3 deletion leads to a dramatic decrease of NANOG level in both ICM and TE in bovine blastocysts. Our mosaic embryo experiments further proved the direct effects of GATA3 in NANOG in the TE, suggesting that TE lineage program is disrupted.

Single embryo RNA-seq analysis reveals 121 genes are differentially expressed in GATA3 KO embryos compared with control embryos. Interestingly, *RhoA* expression level is significantly increased in GATA3 KO embryos (Supplementary Fig. 1F). RhoA is one of the upstream regulators of RhoA/ROCK signaling pathway, which antagonizes TE cell self-renewal and differentiation of bovine preimplantation embryos (Pillai *et al.* 2022), further suggesting GATA3 plays an important role in TE lineage development. Furthermore, GO analysis reveals that DEGs were enriched in cell–cell adhesion, cell–cell junction, and cell morphogenesis involved in cell differentiation. Cell adhesion plays a critical role in the differentiation of the TE (Fleming *et al.* 2001), which interacts with endometrial luminal epithelial cells of the uterus to participate in embryo implantation (Fukuda & Sugihara 2012). Altogether, these results suggest that GATA3 is required for the correct TE lineage program in bovine blastocysts.

In conclusion, we provided evidence that GATA3 and CDX2 regulate cell lineage specification during bovine early embryonic development by using base editors. By comparing with the data in mice, our functional experiments revealed that the interaction of CDX2 and OCT4 differs between mice and cows. Furthermore, the GATA3 mosaic experiment discovered that it is required for correct NANOG expression pattern in bovine TE. We also documented that GATA3 KO disrupted the transcriptome in bovine blastocysts. Our results are helpful for understanding the mechanism behind lineage specification during bovine early embryonic development.

## Supplementary Material

Figure S1. Single blastocyst RNA sequencing of GATA3 and CDX2

Supplementary Material

## Declaration of interest

The authors declare that no conflict of interest could be perceived as prejudicing the impartiality of the research reported.

## Funding

This work was funded by Zhejiang Major Science and Technology Project for Breeding New Agricultural Varieties (No. 2021C02068-1-3 to K.Z.), National Natural Science Foundation of China http://dx.doi.org/10.13039/501100001809 (No. 32072939 to H.W.; No. 31872348, No. 32072731 and No. 32161143032 to K.Z.; No.31941007 to S.W.), Zhejiang Provincial Natural Science Foundation (No. LY19C180002 to H.W.; LZ21C170001 to K.Z.).

## Author contribution statement

Y Shi designed the study and performed microinjection and IF of bovine embryos. B Hu conducted single blastocyst RNA-seq and data analysis. GATA3 and CDX2 sgRNAs were designed by Z Wang. Single embryo PCR and genotyping were conducted by X Wu. *In vitro* production of bovine embryos was performed by Lei Luo and S Li. Y Shi wrote the paper. All other authors reviewed, corrected and approved the final manuscript.
