# Supplementary figures and images for: Functional role of GATA3 and CDX2 in lineage specification during bovine early embryonic development
Source: Reproduction. 2023 Feb 8;165(3):325–33. doi: 10.1530/REP-22-0269 (PMC9986393; doi:10.1530/REP-22-0269)

**A**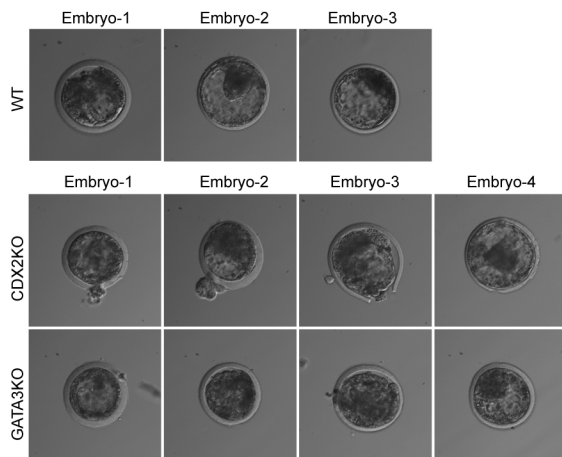**B**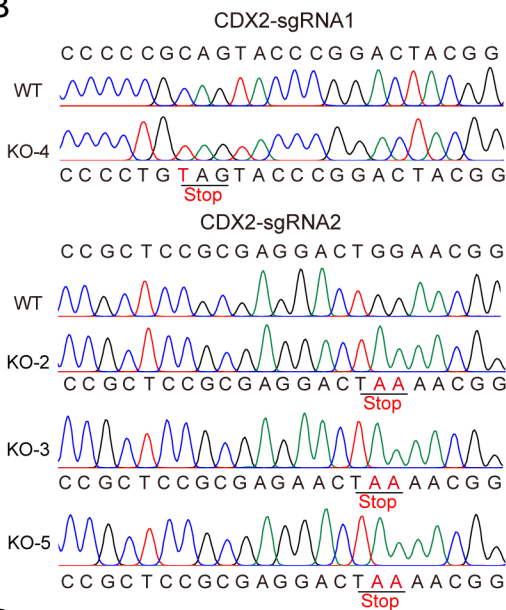**D**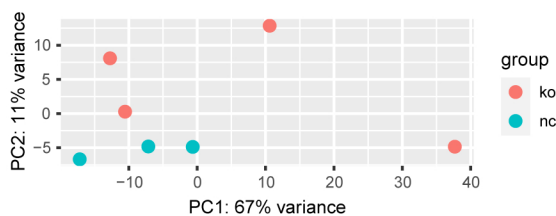**C**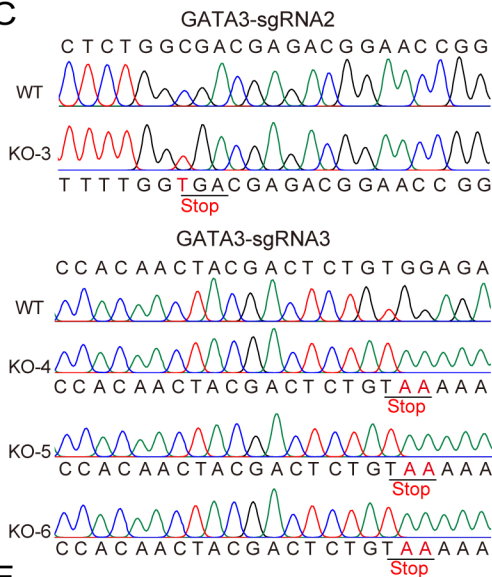**E**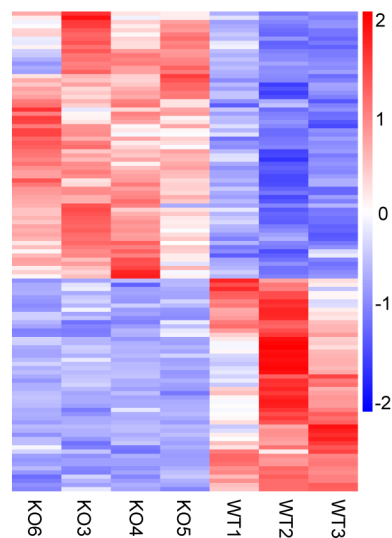**F**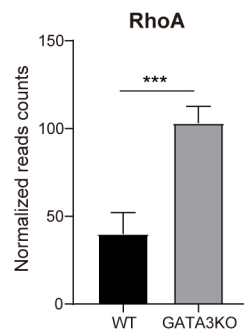

Supplement: Figure S1. Single blastocyst RNA sequencing of GATA3 and CDX2 [file supplementary_figure_1.pdf]
