# Supplementary material for: Functional role of GATA3 and CDX2 in lineage specification during bovine early embryonic development
Source: Reproduction. 2023 Feb 8;165(3):325–33. doi: 10.1530/REP-22-0269 (PMC9986393; doi:10.1530/REP-22-0269)
Supplement: Supplementary Material [file supplementary_material.pdf]

Supplementary Information

Table S1. The synthesis of sgRNAs sequence

Gene name	Gene ID	sgRNA	Exon	Sequence（5' – 3'）
CDX2	618679	sgRNA1	1	FP：CACCCCCCCGCAGTACCCGGACTA<br>RP：AAACTAGTCCGGGTACTGCGGGGG
CDX2	618679	sgRNA2	1	FP：CACCCCGTTCCAGTCCTCGCGGAG<br>RP：AAACCTCCGCGAGGACTGGAACGG
CDX2	618679	sgRNA3	1	FP：CACCTTGCTGCAGACGCTCAACCC<br>RP：AAACGGGTTAGACGTCTGCAGCAA
GATA3	505169	sgRNA1	1	FP：CACCGGCGCAGTACCCTCTGCCCG<br>RP：AAACCGGGCAGAGGGTACTGCGCC
GATA3	505169	sgRNA2	3	FP：CACCCTCTGGCGACGAGACGGAAC<br>RP：AAACGTTCCGTCTCGTCGCCAGAG
GATA3	505169	sgRNA3	4	FP：CACCTCTCCACAGAGTCGTAGTTG<br>RP：AAACCAACTACGACTCTGTGGAGA

Table S2. The primers information of sgRNA template for in vitro transcription

sgRNA	Primers' name	Sequence（5' – 3'）
CDX2-sgRNA1	CDX2-g1-T7-F	TTAATACGACTCACTATAGCCCCCGCAGTACCCGGACTA
CDX2-sgRNA2	CDX2-g2-T7-F	TTAATACGACTCACTATAGCCGTTCCAGTCCTCGCGGAG
CDX2-sgRNA3	CDX2-g3-T7-F	TTAATACGACTCACTATAGTTGCTGCAGACGCTCAACCC
GATA3-sgRNA1	GATA3-g1-T7-F	TAATACGACTCACTATAGGGCGCAGTACCCTCTGCCCG
GATA3-sgRNA2	GATA3-g2-T7-F	TAATACGACTCACTATAGCTCTGGCGACGAGACGGAAC
GATA3-sgRNA3	GATA3-g3-T7-F	TAATACGACTCACTATAGTCTCCACAGAGTCGTAGTTG
	sgRNA-R	AAAAGCACCGACTCGGTGCC

Table S3. Nested PCR primer sequences for preparing Sanger sequencing samples

Gene	Gene ID	Target sgRNAs	Primers' name	Sequence（5' – 3'）
CDX2	618679	CDX2-sgRNA1<br>CDX2-sgRNA2<br>CDX2-sgRNA3	CDX2-g-1	FP：ATGGTGAGGTTCGCCGTC<br>RP：GCTTTACACTGAACGCGGCT

			CDX2-g-2	FP：TACGTGAGCTACCTCCTGGAC<br>RP：CCCCGCTATCCCCTACTCA
		GATA3-sgRNA1	GATA3-g1-1	FP： CCAGTCCTCGCTTCCCTTTT<br>RP：CGCCTGCTGTGACTGTTTTG
			GATA3-g1-2	FP：AATCCTTAAACGACCCCCG<br>RP：CCATGGTGGGTCGGAGGATA
GATA3	505169	GATA3-sgRNA2	GATA3-g2-1	FP： CTGGAGGTGAGAAAGTTCCGT<br>RP：AAGCCAGCTGACACGATTTTG
			GATA3-g2-2	FP：TGCTGGTAGCTTTTTCGTGTG<br>RP：GTCCTGCCAATTTCCTGCAG
		GATA3-sgRNA3	GATA3-g3-1	FP： TACTCCTAGGCCAGTGGGTC<br>RP：TCAAGAAGGGGACCAGGGAA
			GATA3-g3-2	FP：CCGGCCCATGGTCTGATCTAC<br>RP：CACTGGATCCTCCTGCAACG

Table S4：Antibody information

Name	Host	Company	Catalog Number	Application
SOX2	Rat	eBioscience™	14-9811-82	IF (1:200)
CDX2	Mouse	BioGenex	CDX2-88	IF (1:20)
GATA3	Rabbit	Abcam	ab199428	IF (1:200)
GATA6	Rabbit	Cell Signaling Technology	5851T	IF (1:200)
OCT4	Rabbit	Abcam	Ab181557	IF (1:200)
NANOG	Mouse	eBioscience™	14-5768-82	IF (1:100)
SOX17	Goat	Bio-techne	AF1924	IF (1:200)
Alexa Fluor 488	Goat Anti-Mouse	Life technologies	A11001	IF (1:100)
Alexa Fluor 594	Goat Anti-Mouse	Life technologies	A11032	IF (1:100)
Alexa Fluor 594	Donkey Anti-Goat	Life technologies	A11058	IF (1:100)
Alexa Fluor 594	Donkey Anti-Rabbit	Life technologies	A21207	IF (1:100)
Alexa Fluor 488	Goat Anti-Rat	Life technologies	A11006	IF (1:100)
